# Characterization of the Fatty Acyl-CoA Reductase (FAR) Gene Family and Its Response to Abiotic Stress in Rice (*Oryza sativa* L.)

**DOI:** 10.3390/plants13071010

**Published:** 2024-04-01

**Authors:** Danni Zhou, Mingyu Ding, Shuting Wen, Quanxiang Tian, Xiaoqin Zhang, Yunxia Fang, Dawei Xue

**Affiliations:** 1 College of Life and Environmental Sciences, Hangzhou Normal University, Hangzhou 311121, China; zzz20211218@163.com (D.Z.); ding514019@163.com (M.D.); stwen2468@163.com (S.W.); quanxiang@hznu.edu.cn (Q.T.); zxq@hznu.edu.cn (X.Z.); 2 Zhejiang Provincial Key Laboratory for Genetic Improvement and Quality Control of Medicinal Plants, Hangzhou Normal University, Hangzhou 311121, China

**Keywords:** abiotic stress, evolution, FAR, gene expression, rice

## Abstract

Fatty acyl-CoA reductase (FAR) is an important NADPH-dependent enzyme that can produce primary alcohol from fatty acyl-CoA or fatty acyl-carrier proteins as substrates. It plays a pivotal role in plant growth, development, and stress resistance. Herein, we performed genome-wide identification and expression analysis of FAR members in rice using bioinformatics methods. A total of eight *OsFAR* genes were identified, and the OsFARs were comprehensively analyzed in terms of phylogenetic relationships, duplication events, protein motifs, etc. The *cis*-elements of the *OsFARs* were predicted to respond to growth and development, light, hormones, and abiotic stresses. Gene ontology annotation analysis revealed that OsFAR proteins participate in biological processes as fatty acyl-CoA reductase during lipid metabolism. Numerous microRNA target sites were present in *OsFARs* mRNAs. The expression analysis showed that *OsFARs* were expressed at different levels during different developmental periods and in various tissues. Furthermore, the expression levels of *OsFARs* were altered under abiotic stresses, suggesting that *FARs* may be involved in abiotic stress tolerance in rice. The findings presented here serve as a solid basis for further exploring the functions of *OsFARs*.

## 1. Introduction

Plant cuticular waxes constitute an intricate blend of lipids, primarily encompassing very-long-chain fatty acids (VLCFAs) and their derivatives, such as alkanes, primary alcohols, and aldehydes [1,2]. Fatty acyl CoA reductase (FAR) serves as a pivotal enzyme responsible for catalyzing the conversion of very-long-chain (C20:0~C34:0) fatty acids into fatty alcohols [3,4,5,6], playing a crucial role in the biosynthesis of plant cuticles, sporopollenin, and suberin.

Previous studies have shown that different FARs usually exhibit different specificities for acyl chain lengths, resulting in the production of fatty alcohols with different chain lengths that not only affect the composition of plant epidermal wax but are also closely related to plant stress tolerance [7]. The first gene encoding a FAR protein was cloned from *Simmondsia chinensis* [8,9]. Since then, eight *FAR* genes have been identified in *Arabidopsis thaliana* based on the FAR sequence from *S. chinensis* [10,11]. Among them, *AtFAR3*/*CER4* is expressed in various tissues and is mainly responsible for the synthesis of cuticular wax in the epidermal cells in stems. Heterologous expression of *AtFAR3*/*CER4* in yeast can generate C24:0 and C26:0, while the mutation in *CER4* leads to a specific blockade of the alcohol formation pathway [12,13]. Ectopic expressions of wheat (*Triticum aestivum* L.) *TaFAR2*, *TaFAR3,* and *TaFAR4* in the *Arabidopsis cer4* mutant increased the primary alcohol production of C22:0 to C30:0 and partially restored the function of *AtCER4*. In addition, when the transgenic plants were grown under drought conditions, all the *TaFARs* were upregulated, and more leaf cuticular wax was accumulated, which was an important strategy for limiting the dehydration of the plant cells and other organisms under drought conditions [14]. Inhibiting the expressions of *BnA1.CER4* and *BnC1.CER4* in *Brassica napus* can decrease the content of branched primary alcohols and increase the content of other branched components that disrupt the wax crystal type, leading to increased cuticle permeability and enhanced resistance to fungal pathogens [15]. Both poplar (*Populus tomentosa* Carr.) *PtoMYB142* and *Arabidopsis AtMYB94* can bind to the promoter regions of *PtoCER4* and *AtCER4*, respectively, through an abscisic acid (ABA) signaling pathway, which, when induced by drought, results in upregulation of *CER4*, deposition of epidermal waxes, and enhancement of drought resistance [16,17]. *OsFAR1* in rice (*Oryza sativa* L.) responds positively to abiotic stress and ABA induction. Particularly, overexpressing *OsFAR1* can increase the content of primary alcohols and total waxes in *Arabidopsis* and rice, reduce the accumulation of reactive oxygen species (ROS), and enhance drought tolerance in rice [18].

*FAR* also affects plant fertility by regulating lipid metabolism [19]. For example, *AtFAR2*/*MS2* can reduce palmitoyl-ACP in the cytoplasm of tapetum cells to C16:0 alcohols, which are essential primary alcohols in the synthesis of pollen walls. Thus, a loss-of-function mutation in *AtFAR2*/*MS2* leads to abnormal development of the pollen wall [20]. *OsDPW*/*FAR2* from rice and *ZmMS6021* from maize (*Zea mays* L.), highly homologous to *AtMS2*, also exhibit similar functions in the biosynthesis of anther keratin and the development of pollen outer walls [21,22]. With the large-scale completion of plant genome sequencing and the wide application of bioinformatics technology, *FAR* genes have been identified in most species [20,22,23,24,25]. However, no systematic and detailed analysis of the *FAR* family members in rice has been carried out thus far. Therefore, the study of *OsFARs’* responses to abiotic stresses is necessary for enhancing crop tolerance.

In the study, eight members of the rice *FAR* family were identified and their physicochemical properties, motif patterns, gene structures, phylogenetic relationships, *cis*-acting elements, and gene duplication events were comprehensively analyzed. Furthermore, miRNA target prediction and gene ontology (GO) enrichment analysis were also performed. Finally, we investigated the expression patterns of *OsFARs* in different organs/tissues and in response to various abiotic stresses using gene microarray and reverse transcription quantitative real-time polymerase chain reaction (RT-qPCR). This study provides a theoretical framework for understanding the role of *FARs* in the evolution, growth, development, and survival of rice under stresses, and also provides a reference for the subsequent functional verification.

## 2. Results

### 2.1. Whole-Genome Characterization of FARs in Rice

Through comparative analysis and domain identification, eight *OsFAR* genes were identified across the entire rice genome (Table 1). Analysis of physicochemical properties revealed that the lengths of CDS sequences range from 1242 to 1827 bp, and protein lengths range from 413 to 608 aa. The average MW is 56,261.35 Da, ranging from 47,928.54 to 65,228.14 Da, and the pI is between 7.03 and 8.98, with an average of 8.27. Except for OsFAR1 and OsFAR7, the GRAVY values of all other OsFARs are less than 0, indicating that they are hydrophilic proteins. Subcellular localization prediction reveals that OsFAR1 and OsFAR7 reside in the nucleus, OsFAR2 is situated within the chloroplast, and the other OsFARs are located in the cytoplasm.

### 2.2. FAR Protein Structure Analysis

**Figure 1:**
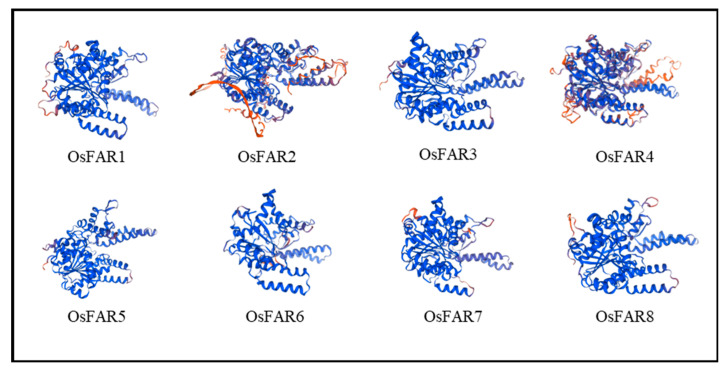
Tertiary structures of OsFAR proteins. Random coils are shown in red.

The secondary structures of the OsFARs were analyzed (Supplementary Table S2). The results indicated that the OsFAR proteins are mainly composed of an alpha helix (42.43~49.31%), random coil (31.09~37.83%), extended strand (14.47~16.46%), and extended helix (4.01~5.3%). The alpha helix and the random coil play a major role in the secondary structure of OsFAR proteins. Differences in gene functions are inextricably linked to their structures [26]. Tertiary structural homology modeling of the OsFAR proteins indicated that all OsFAR proteins, except OsFAR2, are similar (Figure 1), indicating a high level of conservation.

### 2.3. Multiple Sequence Alignment, Phylogenetic Analysis, and Classification of OsFAR Proteins

**Figure 2:**
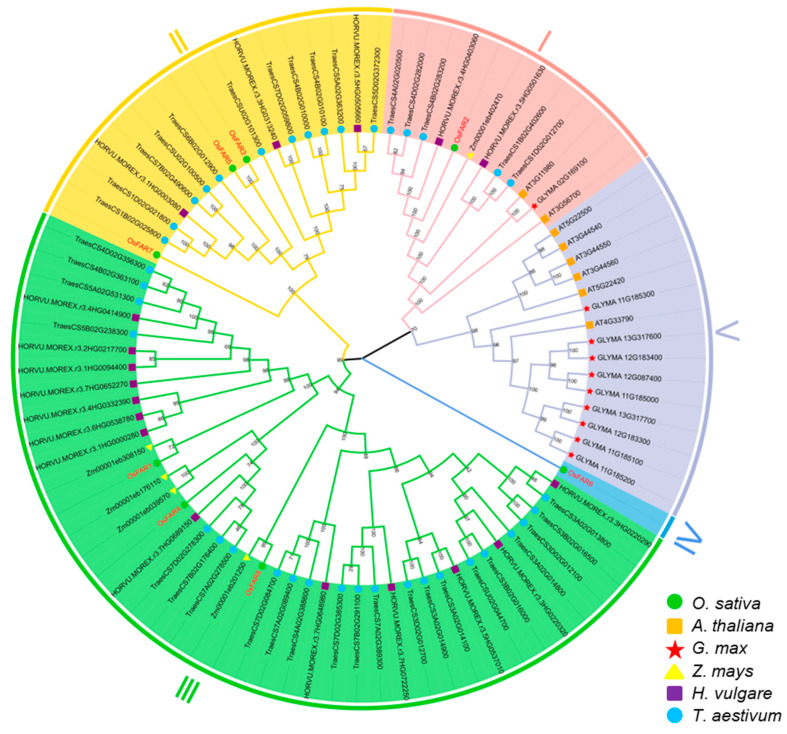
Phylogenetic analysis of the FAR gene family from *Oryza sativa* (Os), *Arabidopsis thaliana* (AT), *Glycine max* (GLYMA), *Zea mays* (Zm), *Hordeum vulgare* (HORVU), and *Triticum aestivum* (Traes). Different colors represent different subfamilies as follows: Group I (red), Group II (orange), Group III (green), Group IV (blue), and Group V (purple).

To explore the evolutionary relationships between the OsFARs, a neighbor-joining (NJ) phylogenetic tree was constructed using protein sequences of FARs from rice, *Arabidopsis*, barley, wheat, maize, and soybean. The results showed that compared to *Arabidopsis* and soybean, the FAR proteins from rice, barley, wheat, and maize exhibited a closer genetic relationship across the entire evolutionary tree (Figure 2). Based on the phylogenetic analysis, the FARs were categorized into five groups (I~V), with each group, except Group V, containing 1~3 OsFARs.

### 2.4. Conserved Motifs and Gene Structures of OsFAR Gene Family Members

**Figure 3:**
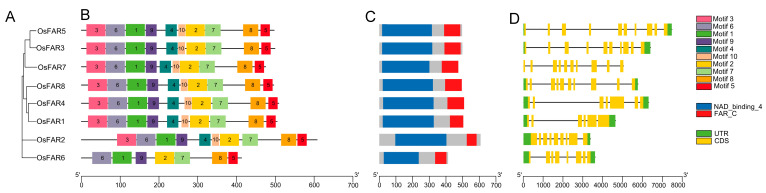
The phylogenetic relationships, conserved motifs, domains, and gene structures of OsFARs. (**A**) Phylogenetic tree of OsFAR proteins. (**B**) Distribution of conserved motifs in OsFARs. The scale bar represents 100 aa. (**C**) Distribution of the NAD_binding_4 and FAR_C domains of OsFARs. (**D**) The structures of the *OsFARs*. Black lines, yellow rectangles, and green rectangles indicate introns, exons, and UTRs, respectively. The scale bar represents 1000 bp.

The analysis of gene structure and conserved motif composition of OsFARs showed that the members belonging to the same subfamily exhibit similar motif structures, especially in terms of the number, type, and arrangement of motifs, which display a significant degree of overlap. Except for OsFAR6, which lacks motifs 3, 4, and 10, all the other OsFARs contain 10 motifs. We also analyzed the protein domains of the OsFARs and found that each OsFAR contains the NAD_binding_4 and FAR_C domains (Figure 3C). Each *OsFAR* gene contains 6~10 exons and 5~9 introns (Figure 3D), and the 5′ UTR and 3′ UTR of *OsFARs* are usually present in the *OsFAR* genomic sequence, with the exception of *OsFAR7*, which lacks both the 5′ UTR and 3′ UTR (Figure 3D). Perhaps due to the specific evolutionary history, function, or genome organization, *OsFAR7* does not have UTR regions, which may affect gene expression regulation, post-transcriptional processing, or mRNA stability [27]. *OsFARs* in related subfamilies often exhibit similar exon/intron structures, indicating a high correlation between phylogenetic relationships among gene family members and gene structure.

### 2.5. Analysis of Cis-Regulatory Element in the Promoter Region of OsFARs

**Figure 4:**
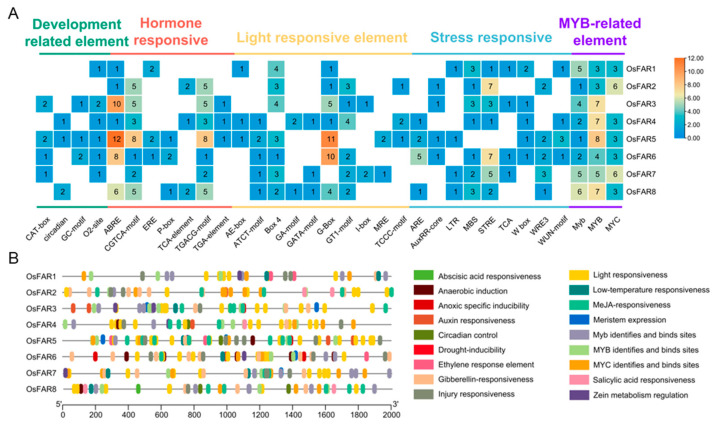
Analysis of CREs in the rice *FAR* promoter region. (**A**) The CREs in the promoters of each *OsFAR* gene. (**B**) The distribution of various CREs in the promoter. Vertical bars with different colors indicate CREs.

*Cis*-regulatory elements (CREs) are located in the promoter region and do not encode proteins but play a crucial role in the regulation of gene expression [28,29]. This study identified a total of 33 major CREs (Figure 4A) that could be categorized into five functional groups: development-related elements, hormone-responsive elements, light-responsive elements, stress-responsive elements, and MYB-related elements. *OsFAR5* had the highest number of CREs (79), whereas *OsFAR1* contained the lowest number (30). All *OsFARs* contained MYB-related elements and light-responsive elements, and most *OsFARs* contained methyl jasmonate (MeJA) elements (TGACG-motif and CGTCA-motif) and ABRE elements (ABA-related) (Figure 4). In addition, 26 stress-response elements (STRE) and 14 drought-response elements (MBS) were found in the promoter region of the *OsFARs*. The above analysis indicates that the *OsFAR* genes may be involved in the regulation of physiological processes such as plant growth and development, light regulation, plant hormone response, and stress.

### 2.6. Analyses of Chromosomal Distribution and Gene Duplication of the OsFAR Genes

**Figure 5:**
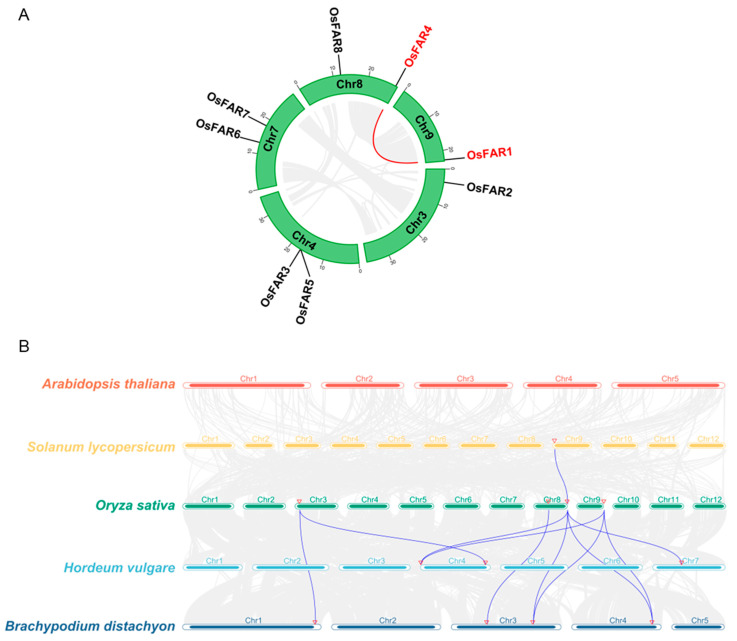
Chromosomal location, collinearity, and evolutionary analysis of *OsFARs*. (**A**) Distribution and collinearity of OsFARs in the rice genome. The collinearity of gene pairs is represented by gray and red lines. Scale bars marked on the chromosomes indicate chromosome lengths (Mb). (**B**) Interspecific collinearity analysis of *FARs* in rice, *Arabidopsis thaliana*, tomato, barley, and *Brachypodium distachyon*. The purple curves show the homology between FAR genes in rice and other species. The red triangles show gene locations.

Based on the genomic data, we found that the *OsFARs* are unevenly distributed on the five chromosomes of rice (3, 4, 7, 8, 9) (Figure 5A), with one each on chromosomes 3 and 9, and two on Chromosomes 4, 7, and 8, respectively.

Gene duplication is crucial for the generation of new genes and functions, and segmental duplication and tandem duplication are important driving forces for the expansion of gene families. Therefore, we analyzed gene duplication events in *OsFAR* genes. The results showed that the eight *OsFARs* are so far away from each other in the genome that no tandem duplication occurs. However, we identified a pair of fragmentally duplicated *FAR* genes (*OsFAR1* and *OsFAR4*) (Figure 5A).

Collinearity analysis can be used to study the evolution and affinity of species [30]. Through collinearity analysis, it was found that there are one pair, four pairs, and six pairs of *FAR* collinear gene pairs between rice and tomato, barley and *B. distachyon*, respectively, but there was no *FAR* collinearity between rice and *Arabidopsis* (Figure 5B). Meanwhile, we found that *OsFAR4* exhibited collinearity in several representative species, except *Arabidopsis*, suggesting that *OsFAR4* is more conserved and plays an important role in the evolutionary process [31].

To further explore the selection of these fragment replication genes, the synonymous mutation frequency (*Ks*) and non-synonymous mutation frequency (*Ka*) values were calculated. The *Ka*/*Ks* ratio provides a powerful tool for elucidating the evolutionary process and selection pressure of *OsFARs* [32], with *Ka*/*Ks* = 1 suggesting neutral selection, values less than 1 indicating purifying selection, and a ratio greater than 1 pointing to positive selection. It was found that the *Ka*/*Ks* values of the segment repeat genes were consistently below 1 (0.445), suggesting that these replication genes were subjected to purifying selection during evolution and were involved in maintaining the conservative structure of the *OsFARs*. We evaluated the *Ka*/*Ks* of collinear gene pairs between rice and other species, resulting in a total of nine collinear gene pairs (Supplementary Table S3), with a range of *Ka*/*Ks* values between 0.178 and 0.306, which suggests that the cross-species FAR family was under purifying or stable selection during evolution.

### 2.7. GO Enrichment Analysis and miRNA Targeting Prediction

The PANNZER online website was used for the GO enrichment analysis of the *OsFARs*. The results (Supplementary Table S4) showed that eight *OsFARs* were significantly enriched for the lipid metabolic process (GO: 0006629). In addition to *OsFAR6*, seven other genes were involved in the suberin biosynthetic process (GO: 0010345). Furthermore, apart from *OsFAR2* and *OsFAR6*, the others were involved in the acyl-CoA metabolic process (GO: 0006637). In terms of molecular function, all eight genes were predicted to be involved in alcohol-forming very long-chain fatty acyl-CoA reductase activity (GO: 0080019) and alcohol-forming long-chain fatty acyl-CoA reductase activity (GO: 0102965). In summary, *OsFARs* are mainly involved in the biological processes associated with lipid metabolism.

**Figure 6:**
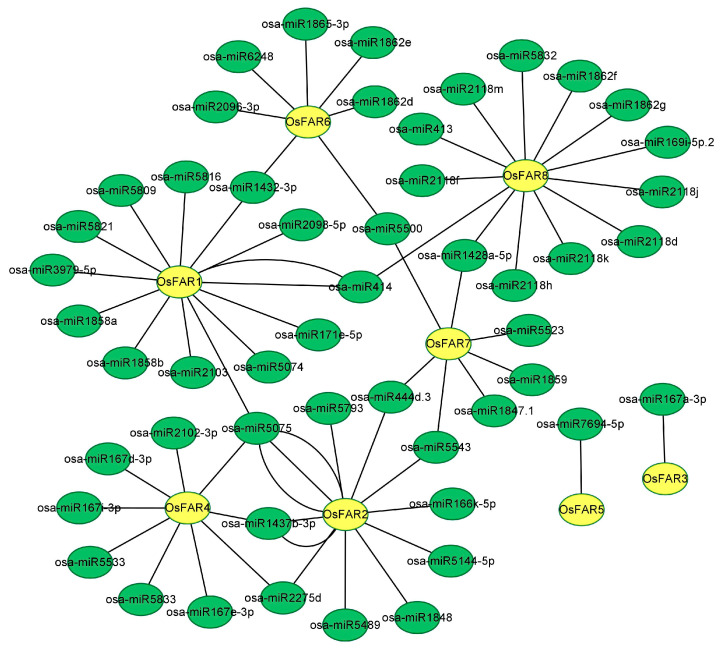
Identification of potential miRNAs targeting *OsFAR* genes.

MicroRNA (miRNA) is an endogenous small-molecule non-coding RNA that mediates the post-transcriptional regulation of gene expression by recognizing and inhibiting target genes through sequence complementation and is also an important regulator of plant growth, reproduction, and stress responses [33]. The CDS sequence of each *OsFAR* was used to predict miRNAs using the psRNATarget database, and a total of 65 mature miRNAs (19–24 nt) were identified (Figure 6). Among these, eight targeted multiple *OsFARs*, while the remaining miRNAs exhibited specificity for each gene. The number of miRNAs targeting *OsFAR8* was the highest (13), while the number of miRNAs targeting *OsFAR3* and *OsFAR5* was the lowest (1). Most predicted miRNAs targeting *OsFAR* had strong regulatory effects through cleavage, while only eight miRNAs regulated *OsFAR* expression through translation inhibition (Supplementary Table S5). It can be inferred that cleavage, serving as the primary function of miRNA, plays a crucial role in regulating the expressions of *OsFARs*.

### 2.8. Expression Patterns of FARs in Different Tissues

**Figure 7:**
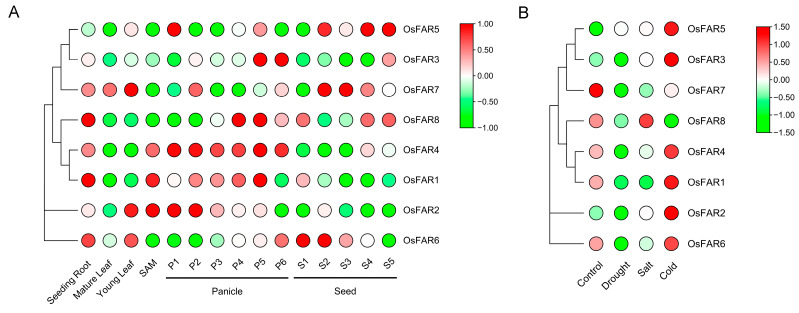
Expressions of *OsFARs* in different tissues and under different abiotic stresses. (**A**) The expression patterns of *OsFARs* in different tissues and developmental stages. The samples were obtained from 7-day-old seedling roots, mature leaves, young leaves, shoot apical meristems, panicles at six stages (P1–P6) divided based on panicle length and days after pollination, and seeds at five distinct stages (S1–S5) classified based on the development stages. (**B**) Expression patterns of *OsFARs* in the shoots of rice seedlings grown under three different abiotic stress conditions. Red indicates a higher expression level while green indicates a lower expression level.

Analyzing the expression patterns of *OsFARs* in different tissues/stages of rice development can further reveal the potential functions of *OsFARs*. Expression data for rice *FARs* in different tissues of the IR64 were obtained. Expression data generated under drought, salt, and low-temperature conditions were also obtained from the Rice eFP Browser website. The results (Figure 7A) indicated that *OsFAR1* and *OsFAR4* exhibited similar expression patterns, with high expression at all stages of young root, SAM, and panicle development, while *OsFAR8* was highly expressed at the P4, P5, and P6 stages of young root and panicle development. Given their unique tissue-specific features, these genes could be crucial for the morphogenesis of the panicle. For example, *OsFAR5*, *OsFAR3*, and *OsFAR2* were highly expressed during a certain stage of panicle development. *OsFAR6* and *OsFAR7* were mainly expressed in young roots and leaves, and at different stages of seed development. However, there was almost no expression of *OsFARs,* except *OsFAR7*, in mature leaves. The results indicated that *OsFARs* exhibit differential expression patterns in various tissues and are potentially involved in panicle development.

To further explore the expression patterns of *OsFARs*, we analyzed microarray data of 7-day-old rice seedlings under three abiotic stresses. As shown in Figure 7B, except for *OsFAR7* and *OsFAR8*, all the *FAR* genes were upregulated under cold stress, while *OsFAR8* expression was upregulated under salt stress. Under drought stress, *OsFAR5* was upregulated, while the other *OsFAR* genes were downregulated. The above results indicate that *OsFARs* are differentially sensitive to different abiotic stresses.

### 2.9. Expression Profiles of OsFARs under Abiotic Stresses

**Figure 8:**
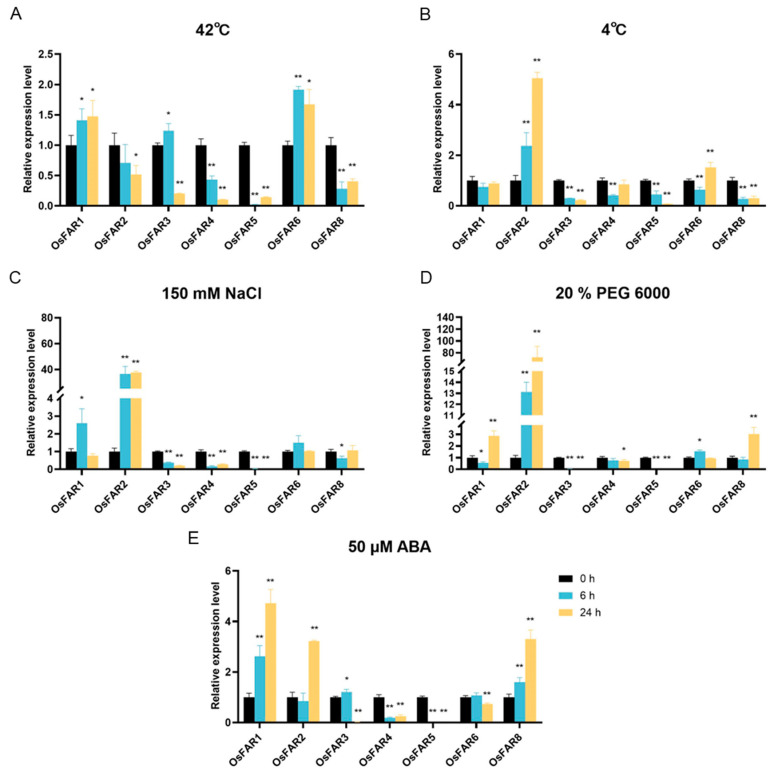
Expression levels of *OsFARs* under different abiotic stresses. (**A**) Treatment at 4 °C. (**B**) Treatment at 42 °C. (**C**) Treatment with 150 mM NaCl. (**D**) Treatment with 20% PEG6000. (**E**) Treatment with 50 μM ABA. All experiments were performed independently at least three times. Error bars represent the standard deviation of replicates. The asterisks (* *p* < 0.05, ** *p* < 0.01, Student’s *t*-test) represent significant differences between the controls and treatments.

In order to study the response patterns of the *OsFARs* under different stress conditions, two-week-old rice seedlings were treated with salt, drought, high temperature, low temperature, and ABA, and samples were taken at 0, 6, and 24 h. The relative expression levels of *OsFARs* in the rice leaves under different stresses were detected using RT-qPCR. The results showed that, except for *OsFAR7*, which was not detected due to its low expression in the leaves, the other *OsFARs* exhibited different responses and regulatory mechanisms under various abiotic stresses (Figure 8).

Under salt, drought, ABA, heat, and cold stress treatments, most *OsFARs* were downregulated compared to the control at most time points. However, excluding heat stress, *OsFAR2* was significantly upregulated by all other stresses and reached a peak at 24 h. The expression of *OsFAR1* was induced by drought, salt stress, and ABA, the same as in previous studies [18]. In addition, *OsFAR6* actively responded to heat stress, while *OsFAR8* actively responded to drought stress and ABA induction. Collectively, these results suggested that *OsFARs* may play a role in responding to environmental stresses. However, the trend of *OsFARs* expression under various abiotic stresses may be inconsistent with the microarray data due to disparities in rice varieties, seedling age, and stress conditions.

## 3. Discussion

Many *FARs* have been identified in plants such as *Arabidopsis* [11,12,13,20,34], wheat [14,24,35], rice [18,21], *B. distachyon* [23,36], and *Gossypium hirsutum* [25]. FARs mediating plant growth, development, and stress response have also been reported. However, it is still important to comprehensively evaluate the genetic characteristics of the FAR family in rice and their role under abiotic stress. Hence, it is crucial to comprehensively characterize the *OsFAR* gene family throughout the entire genome. This is essential for understanding the functional and evolutionary relationships of this gene family in major crop species, thereby enhancing our knowledge of its role in agricultural science.

In the study, we identified eight *FARs* in rice (Table 1) and classified them into five subfamilies (Figure 1). The gene structure and motif distribution of the OsFARs were similar within the same subfamily, indicating that OsFARs in the same subfamily originated from a common ancestor. Genes from a shared ancestor evolved independently at a consistent rate with minimal change. In terms of structural features, all OsFARs contain an NAD_binding_4 domain at the N-terminus, which is involved in their binding to the NAD(P)H cofactor, and a FAR_C domain at the C-terminus that has not yet been attributed a clear role [10]. NAD_binding_4 contains the conserved GXXGXX(G/A) motif and the YXXXK active site motif [14], where tyrosine (Y) and lysine (K) residues are predicted to play a direct role in catalysis [37]. Site-specific mutations of Y and K residues in the YXXXK motif of AtFAR5 prevent yeast from producing primary alcohols [38]. Studies have found that transferring complementary vectors lacking GXXGXX (G/A) or YXXXK motifs into *Arabidopsis ms2* could not restore the defects of the pollen wall, suggesting the essential role of these two conserved motifs in the NAD_binding_4 of MS2 [20]. Subcellular localization prediction showed that OsFAR2 is located in the chloroplast, mainly due to the presence of a transport peptide in its N-terminal region [21] that is observed in other OsFAR2 homologs such as *Arabidopsis* MS2 [20]. OsFAR6 lacks Motifs 3 (related to GXXGXXG motif) and 4 (related to YXXXK motif) (Supplementary Table S6, Supplementary Figure S1). Therefore, it is speculated that *OsFAR6* cannot participate in the normal synthesis of primary alcohols in the fatty acid synthesis pathway, and its detailed biological function needs further exploration.

Gene duplication, mutation, and natural selection are the main sources for the generation of new genes with new functions, providing the basis for biodiversity [39]. Gene duplication significantly contributes to the process of gene amplification. During plant development and growth, gene duplication can help plants adapt to various conditions [40]. Exploring the amplification patterns of gene families can help elucidate their evolutionary processes and functions. Through gene duplication and collinearity analysis, we identified a pair of segmental duplications (*OsFAR1* and *OsFAR4*). OsFAR1 was more closely related to OsFAR4, with 63.48% homology. In addition, the *Ka*/*Ks* ratio of this gene pair was <1, leading us to infer that the *OsFAR* gene family underwent purifying selection during evolution, but the expansion was gradual and highly conserved throughout the evolutionary process. Interspecies collinearity analysis showed that the collinearity between rice and monocotyledonous plants is stronger than that between rice and dicotyledonous plants, which aligns with their kinship distance.

CREs are essential for regulating the spatiotemporal expression of genes, which influences plant growth and development, and coordinates adaptation to the environment. In this study, a total of 23 major CREs were identified, including numerous CREs related to growth and development, hormone response, light response, stress response, and MYB. Among these, most were light-responsive elements (74), followed by jasmonic acid (MeJA)-responsive elements, and ABA-responsive elements. The universal plant hormones MeJA and ABA are widely involved in abiotic stress responses [18,41,42,43]. During wound healing, the exogenous application of ABA could upregulate the expression of *AchnFAR* in kiwifruit and increase primary alcohol accumulation [44]. So far, it has been found that some MYB transcription factors regulate the expression levels of wax-related *FARs* under abiotic stress. For example, *BdMYB41* can directly interact with the promoter region of *BdFAR4*, activating its expression [45]. The synergistic effect of wheat *TaTDRL* and *TaMYB103* can bind to the promoter of *TaTAA1a* and regulate its expression, increasing transcription activity [35]. Many *FARs* have been identified in different species related to plant drought resistance. A total of seven *OsFARs* contain 1~3 drought-responsive CREs, indicating that these genes may be involved in the regulation of drought stress.

The response of plants to external stimuli is regulated by miRNA-mediated gene regulation [46,47]. Previous studies have shown that miRNAs regulate plant growth, development, and stress responses [48,49,50]. For example, the *FAR5* gene in wheat is regulated by miRNA (unconservative_chr5B_part2-30016), which may affect the seed-setting rate in plants [51]. A total of 65 miRNAs were discovered as target genes in the *OsFAR* family, and eight miRNAs specifically target multiple genes. Among these, osa-miR5075 targets three different genes, suggesting that osa-miR5075 may respond to abiotic stresses by regulating the *OsFARs*.

To further understand the potential biological functions of *OsFAR* family members, we analyzed their expression profiles in various tissues/organs of rice and under abiotic stresses using gene microarray data. The results indicated differential expression of *OsFARs* across tissues, suggesting their involvement in panicle development. The accumulation of wax in higher plants is often influenced by various environmental factors, including low temperature, high temperature, and drought. Previous studies have shown that *FARs* can alleviate abiotic stress in rice. Silencing *GhFAR3.1* reduced wax accumulation in upland cotton leaves and weakened drought resistance [25]. The expression of *OsFAR1* was affected by drought stress and it is considered a positive regulator of drought tolerance [18]. Mutations in *BnA1*.*CER4* and *BnC1*.*CER4* resulted in a decrease in wax crystal types and a decrease in the content of branched primary alcohols in *Brassica napus*, thereby reducing the water retention capacity [15]. To discover the roles of *OsFARs* in the response to abiotic stress, data from the gene microarray database and stress treatments were analyzed. The data from the database indicated that *OsFARs* exhibit differential expression under different abiotic stress conditions, with most actively responding to cold stress. The data from the stress treatments also showed that most *OsFARs* were affected by salt, drought, low temperature, high temperature, and ABA. Among these, *OsFAR2* was significantly induced under low temperature, salt, drought, and ABA treatments. Previous research revealed that CREs in the promoter region can be bound by regulatory factors, leading to the induction of their expression under abiotic stress [52]. Our analysis found CREs such as ABREs, MBS, and STREs in the *OsFAR2* gene promoter, indicating its ability to induce *OsFAR2* expression under abiotic stress. Secondly, some miRNAs related to drought responses, such as osa-miR5795 [53], target *OsFAR2* and mediate abiotic stress. Under high-temperature treatment, only *OsFAR1* and *OsFAR6* were upregulated within 24 h. The participation of *OsFAR6* in heat stress response may be related to the regulation of miRNAs such as osa-miR1865-3P [54] and osa-miR1862d [55], which may be involved in heat stress response. Additionally, *OsFAR8* was upregulated by ABA treatment. These results collectively provide a valuable reference for the functional validation of *OsFARs*.

## 4. Materials and Methods

### 4.1. Identification of FARs in Rice

Rice genome, proteome, and related annotation files were obtained from the Ensembl Plants database (http://plants.ensembl.org/index.thml, accessed on 10 March 2023). We downloaded the hidden Markov models (HMM) of the NAD_binding_4 (PF07993) and FAR_C (PF03015) domains from the InterPro website (https://www.ebi.ac.uk/interpro/, accessed on 10 March 2023), and TBtools v2.069 software was used to screen protein sequences containing these domains in rice, with an E-value ≤ 10^−5^ [56,57]. In addition, utilizing the FAR protein sequence of *Arabidopsis* in the search queries, a BLASTP search was executed against the rice database in the Ensembl Plants database to screen potential OsFARs, with the criteria: E-value ≤ 10^−5^ and Identity ≥ 50% [58]. These candidate sequences were then validated for domain composition using both the SMART website (http://smart.embl-heidelberg.de/, accessed on 27 March 2023) and the InterPro website. Candidate genes were named according to previous research and chromosomal locations.

Furthermore, the basic protein characteristics of the OsFARs, including molecular weight (MW), amino acid (aa) length, and isoelectric point (pI) were predicted using the ExPASy website (https://www.expasy.org/, accessed on 2 April 2023) [59]. The subcellular localizations were predicted using the WoLF PSORT website (https://wolfpsort.hgc.jp/, accessed on 2 April 2023).

### 4.2. Prediction of Secondary and Tertiary Structures

To forecast the secondary structures of FARs, the SOPMA website (https://npsa-prabi.ibcp.fr/, accessed on 10 April 2023) was leveraged. Additionally, the tertiary structures of OsFARs were fabricated via homology modeling using the SWISS-MODEL website (https://www.swissmodel.expasy.org/, accessed on 11 April 2023).

### 4.3. Multiple Alignments and Phylogenetic Analysis

To characterize the sequence characteristics of eight OsFARs, multiple sequence alignments were performed using DNAMAN v9 software. To explore the evolutionary relationships between FARs in rice, *Arabidopsis*, barley (*Hordeum vulgare* L.), wheat, soybean (*Glycine max* L.), and maize (*Zea mays* L.), a multiple sequence alignment of FARs from these six species was conducted using the MEGA v7.026 software. Subsequently, a phylogenetic tree of FARs was constructed using the neighbor-joining (NJ) method with the Poisson model. The bootstrap value was set to 1000, with all other parameters set to their default values. Lastly, Evolview (http://www.evolgenius.info/evolview, accessed on 27 April 2023) was employed to visualize the evolutionary tree.

### 4.4. Analysis of Conserved Motifs and Gene Structures

To analyze conserved motifs within the OsFAR proteins, the MEME website (http://meme-suite.org/, accessed on 19 April 2023) was utilized. The default parameters were kept unchanged, except for the number of motifs, which was set to 10 [60]. Functional domain information for the rice FAR family was obtained from NCBI Batch CD-Search (https://www.ncbi.nlm.nih.gov/Structure/bwrpsb/bwrpsb.cgi, accessed on 19 April 2023). The GFF3 (Generic Feature Format version 3 Data) annotation files of *OsFARs* were downloaded from the Ensembl Plants database and visualized using TBtools software.

### 4.5. Cis-Regulatory Element Analysis of OsFARs

In order to explore the CREs in the OsFARs’ promoter region, the TBtools software was used to extract a 2 kb upstream sequence adjacent to each *OsFARs* start codon. Subsequently, these sequences were analyzed using the Plant Care website (http://bioinformatics.psb.ugent.be/webtools/plantcare/html/, accessed on 7 May 2023) and visualized using TBtools [61].

### 4.6. Chromosome Distribution, Gene Duplication, and Selective Pressure Analysis

The genome data for rice, *Arabidopsis*, tomato (*Solanum lycopersicum* L.), barley, and *Brachypodium distachyon* were downloaded from the Ensembl Plants and Phytozome v13 (https://phytozome-next.jgi.doe.gov/, accessed on 16 May 2023) databases [62]. Based on the physical locations of the *OsFARs* in the rice database, the Circos and Dual Synteny Plot tools from TBtools software were used to examine and illustrate the chromosomal distribution, gene duplication events, and gene-level synteny across the various plant genomes. Moreover, the *Ka*/*Ks* ratio was calculated for duplicated gene pairs to assess the selective pressure on genes [63].

### 4.7. GO Functional Annotation and miRNA Targeting Prediction

The PANNZER2 (Protein ANNotation with Z-scoRE, http://ekhidna2.biocenter.helsinki.fi/sanspanz/, accessed on 22 May 2023) tool was used for GO enrichment analysis. The results were visualized using Excel 2010 software [64].

Potential miRNAs targeting *OsFAR* were predicted using the psRNATarget database (https://www.zhaolab.org/psRNATarget/, accessed on 22 May 2023). The CDS sequences of *OsFARs* were used as inputs files and searched against the reference miRNAs in rice. All parameters were kept at default values [65]. The interaction network between the predicted miRNAs and their corresponding target genes was constructed using Cytoscape v3.9.1 software.

### 4.8. Gene Expression Profile Analysis

Expression data for *OsFARs* in 15 different organs/tissues and under 3 different abiotic stress conditions were obtained from the Rice eFP Browser website (https://bar.utoronto.ca/, accessed on 25 May 2023). In addition, the TBtools software was used to visualize the data and generate heatmaps.

### 4.9. Stress Treatment, RNA Extraction, and RT-qPCR Analysis

Seeds from *Oryza sativa* cv. Nipponbare were disinfected with 2.5% NaClO and incubated at 30 °C in darkness. Seedings with consistent growth were selected, transferred to a 96-well PCR plate, and cultivated in a greenhouse under a 14 h day (28 °C) light/10 h (25 °C) night cycle. After two weeks, the seedings were exposed to various stress conditions, including salt (150 mM NaCl), drought (20% (m/V), polyethylene glycol (PEG) 6000), low temperature (4° C), high temperature (42 °C), and the exogenous hormone ABA (50 µM). The aboveground parts of whole plants were grown under various stress conditions and then collected 0, 6, and 24 h after treatment. Rice leaves were then randomly collected, immediately frozen in liquid nitrogen, and stored at –80 °C.

Total RNA was extracted using an Eastern^®^ Super Total RNA Extraction Kit (Promega, Beijing, China), and cDNA synthesis was performed using the Hifair^®^ III 1st Strand cDNA Synthesis SuperMix kit for qPCR (gDNA digester plus) (YEASEN, Shanghai, China) according to the manufacturer’s instructions. RNA was amplified using RT-qPCR using the Hieff^®^ qPCR SYBR Green Master Mix (YEASEN, Shanghai, China). All the primers (Supplementary Table S1) were designed using Primer Premier 5 software, and *OsUBQ5* (LOC_Os01g22490) was used as an internal reference gene. Three biological replicates of each sample were tested, and relative expression levels were calculated using the 2^−ΔΔCt^ method [66]. The expression of *OsFARs* genes was visualized using GraphPad Prism.9.5 software.

## 5. Conclusions

The rice *FAR* gene family consists of eight members that are randomly distributed across five chromosomes. Based on evolutionary analysis, OsFARs can be divided into five subfamilies and include a pair of segmental duplicated genes that may be the way to expand and evolve the *OsFAR* gene family. Members of the same subfamily have similar conserved motifs, gene structures, and protein structures. Inter-species collinearity analysis showed that compared with dicotyledonous plants, OsFARs in rice share greater homology with monocotyledonous plants. The analysis of CREs revealed that *OsFARs* may participate in regulatory pathways such as light, jasmonic acid, abscisic acid, and drought, and the prediction of miRNA targets can help elucidate the mechanism through which miRNAs regulate *OsFARs*. Analysis of gene microarray data revealed that *OsFARs* may participate in rice reproductive growth and actively respond to cold stress treatment. In addition, the results of abiotic stress treatments indicated that the *OsFARs* show differential responses to abiotic stress. A better understanding of the structural and functional characteristics of the *FAR* gene family will be helpful in revealing the regulatory mechanisms underlying plant fatty acid metabolism and provide a theoretical basis for resisting abiotic stress.

## Figures and Tables

**Table 1:** General information on and sequence characterization of eight *OsFAR* genes.

Gene Name	Accession Number		Chromosome	Genomic Location	CDS Length (bp)	Protein				Subcellular Localization
	RAP-ID	MSU-ID				Size (aa)	MV (Da)	pI	GRAVY	
OsFAR1	Os09g0567500	LOC_Os09g39410	9	22660725–22665351	1518	505	56,713.1	8.79	0.044	Nucleus
OsFAR2	Os03g0167600	LOC_Os03g07140	3	3653709–3657069	1827	608	65,228.14	7.03	−0.105	Chloroplast
OsFAR3	Os04g0354600	LOC_Os04g28620	4	16945210–16951596	1500	499	56,729.88	8.53	−0.083	Cytoplasm
OsFAR4	Os08g0557800	LOC_Os08g44360	8	27916313–27922609	1530	509	57,435.52	8.1	−0.082	Cytoplasm
OsFAR5	Os04g0353600	LOC_Os04g28520	4	16882610–16890074	1494	497	56,404.22	7.12	−0.08	Cytoplasm
OsFAR6	NA	LOC_Os07g23340	7	13149738–13153339	1242	413	47,928.54	8.98	−0.249	Cytoplasm
OsFAR7	Os07g0489100	LOC_Os07g30600	7	18106225–18111251	1428	475	53,384.97	8.94	0.017	Nucleus
OsFAR8	Os08g0298700	LOC_Os08g20200	8	12115583–12121337	1491	496	56,266.4	8.67	−0.114	Cytoplasm

## Data Availability

Data sharing does not apply to this article, as no datasets were generated or analyzed during the current study.

## Supplementary Materials

The following supporting information can be downloaded at: https://www.mdpi.com/article/10.3390/plants13071010/s1, Figure S1: Multiple sequence alignment of OsFARs proteins. Table S1: Primers used for qRT-PCR. Table S2: Secondary structure of OsFAR proteins. Table S3: *Ka/Ks* rations of repeating gene pairs within and between OsFAR species. Table S4: The GO annotation and enrichment results of *OsFARs*. Table S5: Predicted miRNA targeting *OsFARs* mRNAs. Table S6: Motif sequences of FARs in rice.
