# Supplementary material for: Characterization of the Fatty Acyl-CoA Reductase (FAR) Gene Family and Its Response to Abiotic Stress in Rice (Oryza sativa L.)
Source: Plants (Basel). 2024 Apr 1;13(7):1010. doi: 10.3390/plants13071010 (PMC11013768; doi:10.3390/plants13071010)
Supplement: Supplementary file 1 [file plants-13-01010-s001.zip › Supplementary Figure.pptx]

## Slide 1
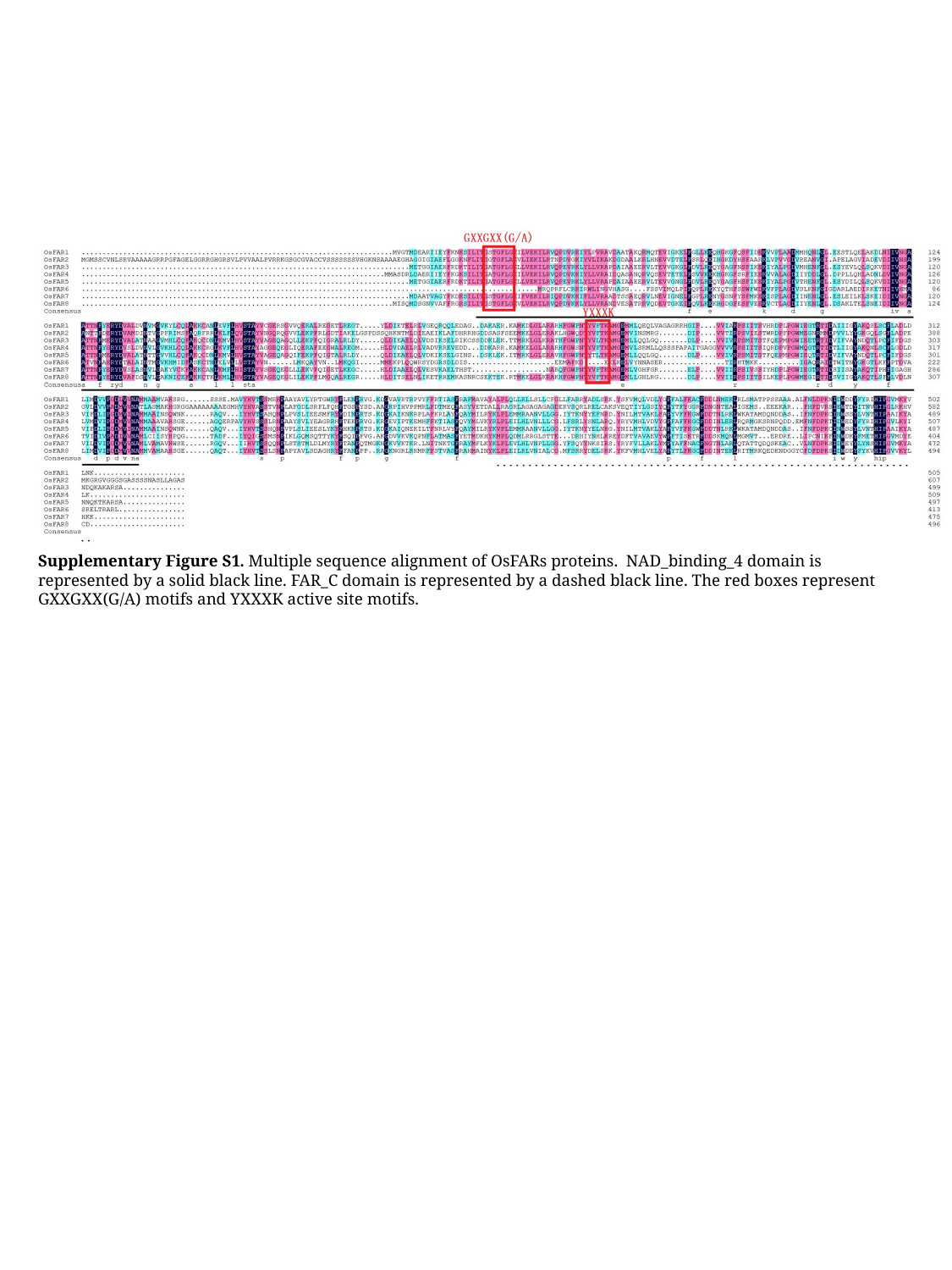

Supplementary Figure S1. Multiple sequence alignment of OsFARs proteins. NAD_binding_4 domain is represented by a solid black line. FAR_C domain is represented by a dashed black line. The red boxes represent GXXGXX(G/A) motifs and YXXXK active site motifs.
